# Analysis between high risk of myocardial infarction with non‐obstructive coronary artery disease in single center and occurrence of major adverse cardiovascular events

**DOI:** 10.1111/anec.13007

**Published:** 2022-10-10

**Authors:** Meihong Kong, Fuzhong Liu, Zhuoxian Zhu

**Affiliations:** ^1^ Special Inspection Branch, The first people's hospital of Jiashan Jiaxing China; ^2^ Department of Cardiovasclar medicine The first people's hospital of Jiashan Jiaxing China; ^3^ Department of third general surgery The first people's hospital of Jiashan Jiaxing China

**Keywords:** major adverse cardiovascular events, myocardial infarction with non‐obstructive coronary artery disease, obstructive coronary artery disease

## Abstract

**Objective:**

To investigate and compare the general information, medication, and the occurrence time of major adverse cardiovascular events (MACE) between patients with myocardial infarction with non‐obstructive coronary artery myocardial infarction (MINOCA) and those with obstructive coronary artery disease (MICAD).

**Methods:**

A total of 325 acute myocardial infarction (AMI) patients were included (MINOCA: *n* = 31; MICAD: *n* = 294). The general information and medication of patients were recorded, including age, gender, prevalence of type 2 diabetes, left ventricular ejection fraction (LVEF), proportion of mitral regurgitation, cTn level, triglyceride level, electrocardiogram (ECG) findings, and drugs used (statins, drugs improving ventricular remodeling, antiplatelet drugs). The above indexes were compared, and statistical analysis was performed at different time points of MACE.

**Results:**

MACE occurred significantly more in the MICAD group than in the MINOCA group (38.8% vs. 12.9%; *p* = .004) after 1 month to 1.5 years of treatment. The earlier the period of MACE occurred in patients with high coronary artery stenosis, it was an independent risk factor for the occurrence of MACE from 1 month to 1 year after surgery (*p* = .002), while the later the occurrence of MACE in patients with LVEF ≥55% (*p* = .029). It was not related to gender, cTn, and electrocardiography (ECG) indexes.

**Conclusion:**

A correlation can be established between the risk factors of MINOCA and the occurrence time of MACE. In addition, MICAD is more commonly seen in male patients and patients with a higher cTn level and lower LVEF.

## Graphical abstract

Compare the general information, medication, and the occurrence time of major adverse cardiovascular events (MACE) between patients with myocardial infarction with non‐obstructive coronary artery disease (MINOCA) and those with obstructive coronary artery disease (MICAD). MACE occurred significantly more in the MICAD group than in the MINOCA group after 1 month to 1.5 years of treatment. The earlier the period of MACE occurred in patients with high coronary artery stenosis, it was an independent risk factor for the occurrence of MACE from 1 month to 1 year after surgery, while the later the occurrence of MACE in patients with LVEF ≥55%.

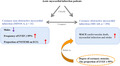

## 1 INTRODUCTION

Acute myocardial infarction (AMI) is usually triggered by coronary artery obstruction and is called MICAD. However, up to 10% of AMI patients show no plaque or thrombosis in coronary angiography (CA) and have no significant obstruction of coronary artery (diameter stenosis <50%); these 10% are named myocardial infarction with non‐obstructive coronary artery disease (MINOCA) (Johnston et al., 2015). MINOCA has been reported to account for 8.6%–13% of AMI patients with an average incidence of 10% (Zhou & Miao, 2019). MINOCA is a group of syndromes with diverse clinical manifestations, and its pathogenesis is varied, including coronary etiologies, such as atherosclerotic plaque rupture, plaque erosion with thrombosis, coronary artery dissection, epicardial coronary vasospasm or microvascular spasm, and coronary embolism, or a combination of mechanisms (Talebi et al., 2021). In the absence of obstructive coronary artery disease, clinicians often fail to diagnose AMI, resulting in wrong treatment (Jung et al., 2021). Therefore, it is important to conduct a thorough assessment of patients.

Major adverse cardiovascular events (MACE) including cardiovascular death, myocardial infarction, and stroke are the primary endpoints for studies on cardiovascular diseases (Li et al., 2020). It has also been shown that MINOCA is still associated with high risk of MACE and poor prognosis, and prognosis is related to the etiologies (Kovach et al., 2021). Therefore, identification of key specific causes is of great significance to the treatment and prognosis of patients, which contributes to the development of targeted treatment with accurate and individualized therapeutic regimen. At present, there are few studies on the correlation between the related factors of MINOCA and MACE. Given this lack, this study collected the clinical data of MINOCA and MICAD patients in First People's Hospital of Jiashan and then selected those that occurred at MACE at different follow‐up time points. We intend to gain an insight into the risk factors for MACE in AMI patients and therefore can conduct early treatment and prevention of intervenable related factors.

## 2 SUBJECTS AND METHODS

### 2.1 Subjects

A total of 325 AMI patients who were hospitalized in the First People's Hospital of Jiashan and underwent coronary angiography (CA) between December 2017 and December 2019 were collected. All patients underwent CA in the subacute phase of myocardial infarction. They were categorized into MICAD and MINOCA patients as per the Fourth Universal Definition of Myocardial Infarction (Thygesen et al., 2018) and the 2016 European Society of Cardiology (ESC) working group position paper (Agewall et al., 2017). MINOCA patients were the research subjects with MICAD patients as controls. The general information of patients was recorded.

Patients over 18 years old who met the diagnostic criteria of AMI were included. Exclusion criteria were as follows: (1) type 3, 4, 5 myocardial infarction; (2) had received thrombolytic therapy before CA; (3) pregnant or lactating women; (4) complicated with severe liver and kidney diseases; (5) had concomitant malignant tumors with an expected survival of less than 1 year. All patients provided informed consents, and this study was approved by the Ethics Committee of the First People's Hospital of Jiashan.

### 2.2 Electrocardiography

All patients underwent routine ECG using a synchronized 12‐lead ECG machine (GE Healthcare) immediately after admission, with a calibration voltage of 10 mm/mV and paper speed of 25 mm/s. Additionally, ST segment amplitude in lead avR was measured with TP segment as the reference level and with the measurement point set at 80 ms after the J point marking the end of QRS. Five consecutive waves should be measured, and the adverse value of 5 times was taken as the change value of ST segment in lead avR. ST segment elevation ≥0.5 mm (0.05 mV) was deemed to be significant (Liu et al., 2016). ECG test was performed by specialized electrocardiographers, and no ST‐T changes, ST‐segment elevation myocardial infarction (STEMI), and non‐ST‐segment elevation myocardial infarction (NSTEMI) were distinguished. ECG images of STEMI are characterized by ST‐segment arcuate back elevation, new complete left bundle branch block, or hyperacute T waves; ECG images of NSTEMI present with new ST‐segment depression, and occasionally present with ST‐segment depression in multiple leads or T‐wave flatness or inversion (Yan & Lu, 2015).

### 2.3 Outcome measures

General data of patients were collected, including gender, age, cases of mitral regurgitation, cases of type 2 diabetes, cardiac troponin (cTn) level, triglyceride level. Drug applications were also recorded, including statins, β‐blockers, angiotensin‐converting enzyme inhibitors/angiotensin II receptor blockers (ACEI/ARB), antiplatelet drugs (aspirin and clopidogrel).

According to the ECG parameters of the two groups, left ventricular ejection fraction (LVEF) was collected. LVEF can reflect the left ventricular systolic function; good LVEF was in a range of 55%–60% and classified as LVEF ≥55% in statistical analysis in this study.

### 2.4 MACE diagnosis

The occurrence of MACE during hospitalization and 1.5 year follow‐up was recorded, including recurrent angina pectoris, new heart failure, malignant arrhythmia, reinfarction, cardiogenic shock, and cardiac death (Yan & Lu, 2015). Incidence of MACE = (the number of patients with MACE/total number of patients) x 100%.

### 2.5 Statistical analysis

SPSS 23.0 statistical software was applied for result analysis. Measurement data conforming to normal distribution were expressed as mean ± standard deviation (SD), and statistical difference between means of two groups was tested by independent sample t‐test; measurement data failing to meet normal distribution were expressed as median (Q1, Q3). For categorical data, chi‐square test was used for that in normal, and rank sum test for that in non‐normal distribution (Z value was adopted). *p* < .05 was considered statistically significant.

## 3 RESULTS

### 3.1 General information

The general data of patients are shown in Table 1. A total of 325 AMI patients were included (MINOCA: *n* = 31; MICAD: *n* = 294). No significant difference was identified in age between the two groups. Compared with the MICAD group, MINOCA group had less males, higher LVEF levels, higher frequency of LVEF ≥55%, lower cTn level, less frequency of chest or admomen pain at most 12 hours and higher proportion of NSTEMI on ECG test. Additionally, the MINOCA group had fewer cases of mitral regurgitation, a lower prevalence of diabetes, and higher triglyceride levels, but the difference was not statistically significant. Eighty‐five MICAD patients belonged to neither NSTEMI nor STEMI.

**TABLE 1:** General information of patients

Basic information	MINOCA group (*n* = 31)	MICAD group (*n* = 294)	*p*
Age (year)	65.55 ± 1.91	65.65 ± 0.69	.963
Gender(males, %)	22 (71.0%)	224 (76.2%)	**<.01**
cTn (ng/ml)	2.72 ± 1.80	8.06 ± 1.00	**.007**
LVEF ≥55%(%)	29 (93.5%)	231(78.6%)	**<.011**
LVEF(%)	64.00 ± 1.77	59.63 ± 0.54	**.011**
Mitral regurgitation	10 (32.3%)	131(44.6%)	.189
Electrocardiography			**<.01**
Proportion of NSTEMI	20(61.2%)	85(28.9%)	
Proportion of STEMI	11(38.7%)	134(45.6%)	
Proportion of no ST‐T changes	0	75(25.5%)	
History of type 2 diabetes	3(9.7%)	58(19.7%)	.173
Triglyceride (mmol/l)	1.58 ± 0.32	1.49 ± 0.06	.656
Time before operation ≤12 h	6(19.4％)	100(34％)	.098

*Note*: Data are presented as Mean ± SD or *n* (%).

Abbreviations: cTn, cardiac troponin; LVEF, left ventricular ejection fraction; NSTEMI, non‐ST‐segment elevation myocardial infarction; STEMI, ST‐segment elevation myocardial infarction.

### 3.2 Comparison of medication and MACE in MINOCA patients

We further compared the medication and the occurrence of MACE between the two groups. As shown in Table 2, the proportion of all types of drugs used was significantly higher in MICAD patients than in MINOCA patients (*p* < .05). At different time periods after treatment (<1 month, 1 month–1 year, 1 year–1.5 years, >1.5 years), a significant difference was identified in the occurrence of MACE between MINOCA patients and MICAD patients (*p* < .05). After 1 month to 1.5 years of treatment, MACE occurred in 4 (12.9%) MINOCA patients and 114 (38.8%) MICAD patients. Generally speaking, more MICAD patients were attacked by MACE compared to MINOCA patients.

**TABLE 2:** Comparison of medication and MACE observed at different time points of the two groups

Drugs	MINOCA group (*n* = 31)	MICAD group (*n* = 294)	*p*
Statins	26(83.9%)	287(97.6%)	**<.01**
Drugs improving ventricular remodeling			**.003**
β‐blocker combined with ACEI/ARB	3 (9.7%)	62 (21.1%)	
β‐blocker	4(12.9%)	95(32.3%)	
ACEI/ARB	9 (29.0%)	54 (18.4%)	
No drug use	5(16.1%)	6(2.0%)	
Antiplatelet drugs			**.002**
Dual antiplatelet therapy	25(80.6%)	279(94.9%)	
Aspirin or clopidogrel used alone	1(3.2%)	9(3.1%)	
No drug use	5(16.1%)	6 (2.0%)	
MACE occurring at different time points			**.004**
<1 month	1(3.2%)	12(4.1%)	
1 month‐1 year	0	50(17.0%)	
1 year‐1.5 years	3(9.7%)	52(17.7%)	
>1.5 years	27(87.1%)	180(61.2%)	

Abbreviations: ACEI/ARB, angiotensin‐converting enzyme inhibitors/angiotensin II receptor blockers; Data, n (%); MACE, major adverse cardiovascular events.

### 3.3 Risk Factors of MACE in AMI Patients

AMI patients were grouped according to the different time points of MACE and analyzed the risk factors of MACE in AMI patients by logistics multivariate regression. As shown in Table 3, in patients with no MACE within 1.5 years after treatment as controls, the degree of stenosis in AMI patients with MACE was higher. Additionally, patients with MACE who occurred between postoperative 1 month and 1 year displayed the most severe stenosis compared to the controls, with a partial regression coefficient of 0.034. The above results showed that a high degree of coronary stenosis was an independent risk factor for MACE occurred between postoperative 1 month and 1 year (*p* < .05).

**TABLE 3:** Logistics multivariate regression analysis of MACE occurrence at different time periods (with no MACE within 1.5 years as control)

Basic information	MACE occurred within 1 month (*n* = 13)	MACE occurred between 1 month and 1 year (*n* = 50)	MACE occurred between 1 year and 1.5 years (*n* = 55)	*p*
	OR (95%CI)	OR (95%CI)	OR (95%CI)	
Gender(male)	0.858 (0.220, 3.340)	0.645 (0.315, 1.320)	0.888 (0.434, 1.818)	>.05
cTn	0.989 (0.952, 1.028)	0.998 (0.978, 1.018)	1.006 (0.998, 1.025)	>.05
Degree of stenosis (%)	1.030 (0.991, 1.069)	1.034 (1.013, 1.056)	1.014 (0.998, 1.030)	**.002**
Electrocardiogram				
NSTEMI	0.535 (0.130, 2.191)	0.850 (0.405,1.783)	0.512 (0.232, 1.130)	>.05
No ST‐T changes	0.833 (0.198, 3.507)	0.966 (0.416, 2.244)	1.365 (0.656, 2.843)	>.05
LVEF≥55%	0.874 (0.249, 3.065)	2.827 (1.112, 7.189)	4.861 (1.641, 14.392)	**.029**

Abbreviations: cTn, cardiac troponin; LVEF, left ventricular ejection fraction; MACE, major adverse cardiovascular events; NSTEMI, non‐ST‐segment elevation myocardial infarction.

**FIGURE 1:**
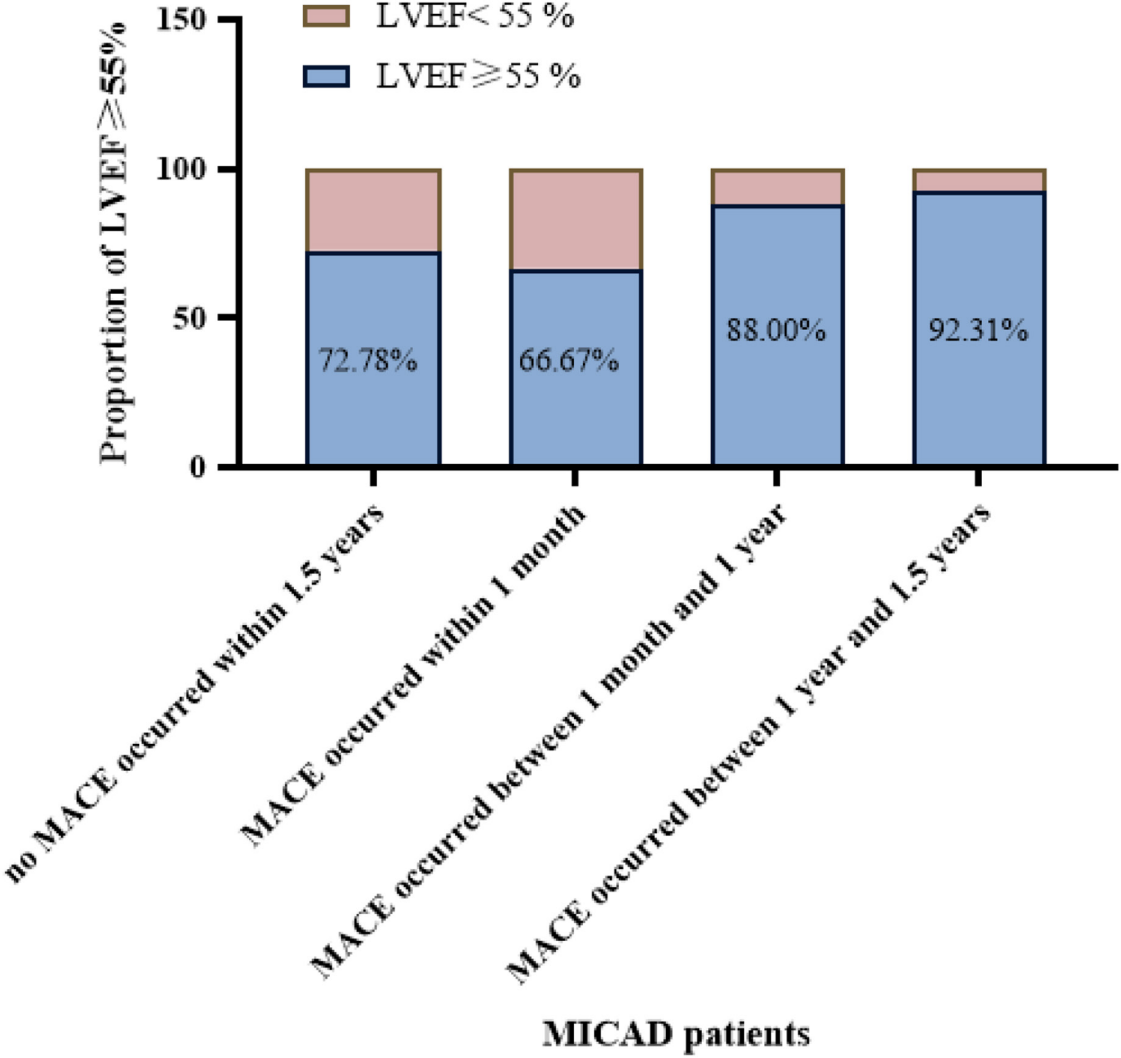
The relationship of MACE in MICAD patients with left ventricular systolic function. (LVEF, left ventricular ejection fraction; MACE, major adverse cardiovascular events)

In addition, during 1.5 year follow‐up, we found the later the occurrence of MACE, the higher the proportion of LVEF ≥55%. According to Figure 1, for MICAD patients with more than 50% coronary stenosis, the later the occurrence of MACE, the more patients show good systolic function. Collectively, we estimated that good cardiac function was associated with delayed occurrence of MACE.

## 4 DISCUSSION

The pathogenesis of MINOCA may be related to hypertension, aortic stenosis, coronary artery spasm, coronary thromboembolism, spontaneous coronary dissection, coronary microvascular dysfunction, stress cardiomyopathy, coronary plaque rupture, type 2 acute myocardial infarction (myocardial cell necrosis caused by an imbalance in myocardial oxygen supply and demand), or unknown etiology (Bairey Merz et al., 2017). After sudden coronary artery infarction attacks, patients are found with ischemia in the blood supply area, damaged myocardial cells, and severe ion leakage. In the ischemic area, the transmembrane potential near the endomyocardium is close to normal, while that in the damaged area is low. This potential difference results in a dome‐shaped ST segment elevation. Therefore, the change of ST segment in ECG can help shed light on whether the coronary artery is recanalized after CA (Wei et al., 2020). ECG after admission serves as a test to obtain direct information on acute myocardial ischemia in the early stage and to assess risk stratification of patients in the acute phase. The MINOCA patients included in this study were mainly NSTEMI, while STEMI accounted for a relatively large part of MICAD patients. Previous studies also found that the proportion of NSTEMI patients in the MINOCA group was 65.7%, which was much higher than that in the AMI group (40.9%) (Jiang et al., 2020).

We also found that compared with the MICAD group, the MINOCA group had less males, higher LVEF level and a higher proportion of LVEF ≥55%, and lower cTn levels, which the 55% is considered to be the threshold for whether clinical LVEF is normal. Therefore, we used LVEF≥55% as the cut‐off point for both groups, and we also found a later onset of MACE in patients with LVEF≥55%. In addition, the proportion of mitral regurgitation in MINOCA patients was less, but not significantly different from that in the MICAD group. Some studies have stated that STEMI patients are predominantly male (Ruiz Pizarro et al., 2019), and NSTEMI patients show lower mitral early‐diastolic flow peak velocity than in normal patients (Cheng et al., 2011). ST‐segment elevation and reduced LVEF are closely related to mitral regurgitation (Lissin et al., 2002; Nabati et al., 2016). Henceforth, the reason why there is no significant difference in the proportion of mitral regurgitation between the two groups may be related to the interference of potential confounding factors. cTn is a complex of three subunits, namely cTnC, cTnI, and cTnT, and is considered to be the most specific biochemical marker currently used for the diagnosis of acute coronary syndrome. It has been reported that cTn is lower in MINOCA patients than in MICAD patients and thus can predict the prognosis of MINOCA patients (Hjort et al., 2018). This is consistent with the findings of our study.

Some studies have found that triglyceride levels in patients with NSTEMI are significantly higher than those in patients with STEMI and unstable angina pectoris (Yang et al., 2021). Similarly, MINOCA group had higher triglyceride levels compared with the MICAD group in our study, but the difference was not statistically significant, and the reason may be the small number of samples included in the MINOCA group. In addition, we found that lipid metabolism was improved by the use of statins. The proportion of statins used in MINOCA patients was markedly lower than that in MICAD patients. Collectively, for MINOCA patients, the use of triglycerides and statins needs further attention.

The criteria for MACE refer to the standard published in 2021 from 97 hospitals in China: (A) primary endpoints: cardiac death, non‐fatal myocardial infarction, or non‐fatal stroke; (B) secondary endpoints: all‐cause mortality, non‐fatal myocardial infarction, non‐fatal stroke, hospitalization for unstable angina, or heart failure, and coronary angioplasty (percutaneous coronary intervention or coronary artery bypass grafting) (Ge et al., 2020). Retrospective analysis of in‐hospital MACE pointed out that the proportion of MACE in MINOCA patients was similar to that in MICAD patients, showing no significant difference between the two (Nordenskjöld et al., 2018; Núñez‐Gil et al., 2008). However, it has been found that the incidence of one‐year all‐cause mortality and non‐fatal myocardial infarction was 3.2% and 8.3%, respectively, in MINOCA patients, and 4.2% and 7.9%, respectively, in MICAD patients (Williams et al., 2019). Therefore, the prognosis of MINOCA has greater potential to be improved than that of MICAD. In this study, we observed MACE within 1 month in patients and found no significant difference in the incidence of MACE between MINOCA patients and MICAD patients. After 18 months of follow‐up, the incidence of MACE was found to be significantly lower in MINOCA patients than in the MICAD group. In particular, the incidence of MACE in MICAD from 1 year to 1.5 years after treatment remained high and was similar to that within 1 year after treatment. Hence, it can be concluded that a high degree of coronary stenosis is an independent risk factor for MACE in AMI patients from 1 month to 1.5 years after treatment.

Antiplatelet and anti‐ischemic therapy are recommended for patients with acute coronary syndrome (Association et al., 2016). Studies have found that patients in north China suffer from MACE, type 2, 3, and 5 bleeding defined by the Bleeding Academic Research Consortium during 5 years follow‐up more frequently than patients in the southern region, which may be associated with the low compliance of dual antiplatelet or single antiplatelet for secondary prevention in northern patients during 1–5 years of follow‐up (Wang et al., 2021). In our study, the proportion of antiplatelet medications for secondary prevention in MINOCA patients was small, possibly due to inadequate attention paid to the degree of stenosis.

This study is limited to some extent. First, the possibility of dropouts of systolic blood pressure, heart rate, body mass index, creatinine, smoking, vascular disease, and other factors could not be ruled out. Second, the study was a single‐center study, not combined with other multi‐center large‐sample clinical trials, which led to the small sample size. Finally, no intervention trial was conducted after treatment for all patients.

## 5 CONCLUSION

MINOCA group has fewer male patients than MICAD patients. Besides, compared with the latter, MINOCA patients have relatively higher LVEF levels, higher proportion of LVEF ≥55%, and lower cTn levels; they manifest NSTEMI on the ECG. After surgery, less attention has been paid to the use of statins, drugs that improve ventricular remodeling, and antiplatelet drugs in MINOCA patients. During 1.5 year follow‐up, MINOCA patients have less MACE because of less organic injury to the affected coronary artery.

## AUTHOR CONTRIBUTIONS

Meihong Kong and Fuzhong Liu designed this work. Fuzhong Liu and Zhuoxian Zhu performed the data extraction and statistical analyses. Meihong Kong and Zhuoxian Zhu wrote this article. All authors read and approved the final manuscript.

## FUNDING INFORMATION

This study was supported by Clinical Research Fund Project of Zhejiang Medical Association (No. 2019ZYC‐A38).

## CONFLICT OF INTEREST

The authors declare that they have no competing interests.

## ETHICAL APPROVAL

All patients provided informed consent, and this study was approved by the Ethics Committee of the First People's Hospital of Jiashan.

## CONSENT FOR PUBLICATION

Not applicable.

## Data Availability

The data that support the findings of this study are available from the corresponding author upon reasonable request.
